# Sex and age differences in the association between heart rate variability and cardiac chronotropy: A replication‐extension study

**DOI:** 10.14814/phy2.70399

**Published:** 2025-06-10

**Authors:** Luca Carnevali, Darcianne K. Watanabe, Margherita Barbetti, Suzi Hong, DeWayne P. Williams, Jordan Kohn, Julian Koenig, Julian F. Thayer

**Affiliations:** ^1^ Department of Chemistry, Life Sciences and Environmental Sustainability University of Parma Parma Italy; ^2^ School of Social Ecology, c/o Department of Psychological Science University of California Irvine, 4201 Social and Behavioral Sciences Gateway Irvine California USA; ^3^ Herbert Wertheim School of Public Health & Human Longevity Science University of California San Diego La Jolla California USA; ^4^ Department of Psychiatry, School of Medicine University of California San Diego La Jolla California USA; ^5^ Department of Psychological Science University of California Irvine, 4201 Social and Behavioral Sciences Gateway Irvine California USA; ^6^ Department of Child and Adolescent Psychiatry, Psychosomatics and Psychotherapy, Faculty of Medicine University of Cologne, and University Hospital Cologne Cologne Germany

**Keywords:** aging, heart rate variability, hormone replacement therapy, sex differences

## Abstract

Using heart rate variability (vmHRV) as a proxy of cardiac vagal modulation, previous studies have hinted at sex differences in the vagal control of cardiac chronotropy in young adults, but little is known in older individuals. The current study aimed at investigating for the first time the moderating role of both sex and age in the relationship between vmHRV and cardiac chronotropy in younger (*n* = 106, mean age: 19.9 (3.5) years) and older (*n* = 109, mean age: 72.8 (2.6) years) individuals. Further, we explored the effects of hormone replacement therapy on such association in a sub‐sample of post‐menopausal women (*n* = 17). Resting measures of the average inter‐beat interval (IBI, as index of cardiac chronotropy) and vmHRV were collected. The results indicate (i) stronger associations between vmHRV and IBI in young adults and post‐menopausal women compared to age‐matched men, (ii) a weaker or no association in older women and men, respectively, and (iii) no effects of hormone replacement therapy in post‐menopausal women. This study provides evidence of sex and age differences in the association between vmHRV and cardiac chronotropy, offering novel insight into vagal mechanisms of cardiac chronotropic control that may inform our understanding of sex‐ and age‐related vulnerability to negative health outcomes.

## 1 INTRODUCTION

Regulation of cardiac rhythm is complex and occurs at least at two major levels: the intrinsic “coupled‐clock” system within the sinoatrial node, the cardiac pacemaker, and the autonomic nervous system (ANS) (Behar & Yaniv, 2016; Tsutsui et al., 2018). Mutual entrainment between intrinsic and ANS mechanisms primarily determines the average interbeat interval (IBI) for a segment of time and IBI variations from beat to beat. The latter can be quantified via heart rate variability (HRV) analysis, which provides several metrics that can be reliably utilized as proxies of cardiac vagal modulation (Laborde et al., 2017). A well‐known association exists between vagally mediated (vm)HRV indices and IBI such that lower vmHRV is associated with shorter IBI (i.e., faster heart rate) and vice versa. Importantly, we recently demonstrated that sex moderates the relationship between vmHRV and IBI both in young adult humans (Williams et al., 2022) and rats (Carnevali et al., 2023). Specifically, women and female rats showed significantly stronger correlations between vmHRV and IBI (Carnevali et al., 2023; Williams et al., 2022). Also, at any given level of vmHRV, the average IBI was shorter in young adult women and female rats compared to men and male rats (Carnevali et al., 2023; Williams et al., 2022). These findings support meta‐analytic evidence showing that women have shorter IBI (i.e., faster heart rate) than men (Koenig & Thayer, 2016), yet at any given level they have higher vmHRV. This suggests the existence of important sex differences in the vagal modulation of cardiac chronotropy. For example, vagal stimulation elicits release of acetylcholine (ACh), and converging evidence suggests greater ACh sensitivity in females compared to males (Dart et al., 2002; Pinto et al., 1997; Sarabi et al., 1999; Taddei et al., 1996), which may be due to the prevailing effects of male and/or female sex hormones. Such prevailing hormone levels may also produce differences between pre‐ and post‐menopausal women. Further, human and animal studies have documented an age‐related decline in HRV (Abhishekh et al., 2013; Piantoni et al., 2021), which has been reported as a predisposing factor for the development of psychiatric and somatic (e.g., cardiovascular) disorders (Brown et al., 2018; Greiser et al., 2005; Tsuji et al., 1996; D. K. Watanabe, M. N. Jarczok, D. P. Williams, J. Koenig, & J. F. Thayer, unpublished results). However, the implications of this age‐related HRV reduction on vagal mechanisms of cardiac chronotropic control are still elusive. Thus, an investigation to examine the influence of both age and sex in the association between vmHRV and cardiac chronotropy would reveal the importance of considering key demographic characteristics in understanding cardiac control mechanisms, as previous studies included only young adult individuals. This may provide novel insight into individual vulnerability to psychophysiological outcomes.

**FIGURE 1:**
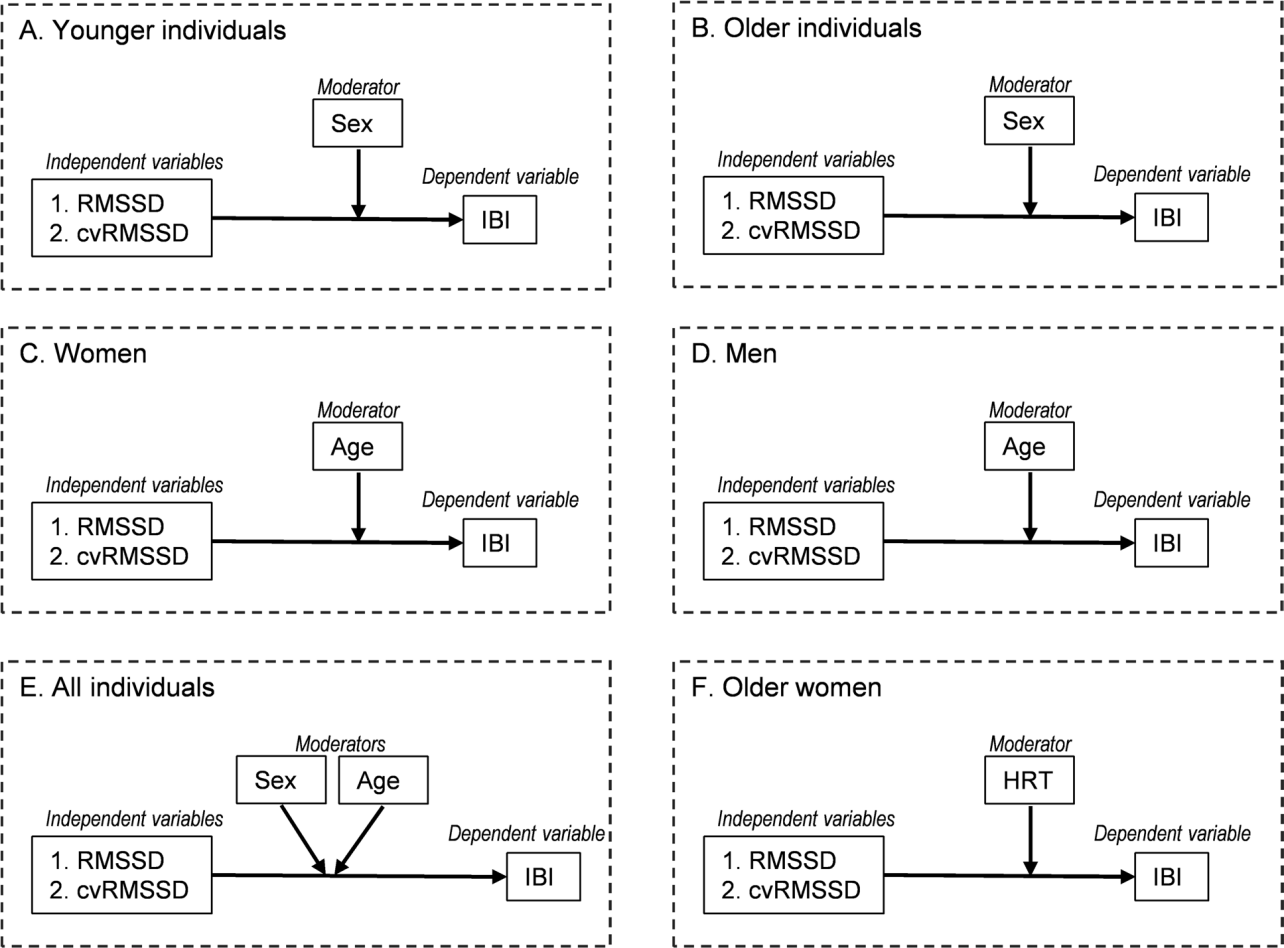
Conceptual moderation models of the current investigation. cv, coefficient of variation; HRT, hormone replacement therapy; IBI, inter‐beat‐interval; RMSSD, root mean square of successive in beat‐to‐beat interval differences.

Based on these considerations, the main objective of the current study was to investigate the moderating role of both sex and age in the association between vmHRV and IBI (see Figure 1 for conceptual models). We aimed at replicating previous findings of sex differences in a new sample of younger adults, and at extending this investigation for the first time to post‐menopausal women vs. age‐matched men. Next, we tested the moderating role of age in the association between vmHRV and IBI within each sex. Further, we explored the effect of hormone replacement therapy (HRT) with estrogen/progesterone on such association in post‐menopausal women. In all models, we adopted the root mean square of successive beat‐to‐beat interval differences (RMSSD) as the vmHRV index. There is an ongoing debate on whether HRV metrics should or should not be corrected for the heart period, for example, by calculating the coefficient of variation (cv) of HRV (de Geus et al., 2019). In the previous study, weaker associations were found between HRV measures and indexes of cardiac chronotropy when HRV variables were “adjusted” for IBI (Williams et al., 2022). To corroborate those findings, in the current analyses we included both “unadjusted” RMSSD values and “adjusted” cvRMSSD values.

## 2 MATERIALS AND METHODS

The present investigation is based on data collected from two samples of healthy young adults (48 women, 58 men; mean age (SD) = 19.9 (3.5) years) and older individuals (76 women [17 on HRT], 33 men; mean age (SD) = 72.8 (2.6) years) with managed hypertension. In the older sample, 68 of 109 (62.4%) participants were taking at least one cardiovascular medication. Medications included beta‐blockers (*n* = 29, 26.6%), diuretics (*n* = 19, 17.4%), vasodilators (*n* = 4; 3.7%), angiotensin II receptor blockers (*n* = 26, 23.9%), calcium‐channel blockers (*n* = 22; 20.2%), and angiotensin‐converting enzyme inhibitors (*n* = 19, 17.4%). Of note, these percentages do not add to 100% as the criteria for the older sample were that participants were people with controlled hypertension. Thus, participants may have been taking multiple drugs, a regimen necessary to achieve blood pressure control. Participants were recruited at The Ohio State University (sample 1) and UC San Diego (sample 2). Another study investigated blood pressure and related measures obtained from the same samples but did not assess the relationships between IBI and HRV data (D. K. Watanabe, M. N. Jarczok, D. P. Williams, J. Koenig, & J. F. Thayer, unpublished results).

Protocols were approved by the Ohio State University Human Subjects Institutional Review Board and UC San Diego Institutional Review Board (#150953), and all participants signed written informed consent at the respective study sites. Available demographic variables were sex, age, and body mass index (younger females = 24.4 ± 4.2 kg/m^2^; younger males = 25.1 ± 4.2 kg/m^2^; older females = 28.2 ± 6.2 kg/m^2^; older males = 29.6 ± 5.7 kg/m^2^). Participants were asked to refrain from exercise, caffeine, and smoking for 12 hours before the experiment, which took place for both samples in a quiet dimmed room. Participants' successive IBIs (in milliseconds) were recorded from the non‐dominant middle finger during a 5‐min resting period by using the Finometer Pro (Finapres Measurement Systems, Amsterdam, The Netherlands).

### 2.1 Cardiac measures

IBIs were analyzed using Kubios HRV analysis package 2.0 (Tarvainen et al., 2014). Artifacts within the IBI series were removed using a threshold‐based correction based on a piecewise cubic interpolation method (Tarvainen et al., 2014). From the IBI series, the root mean square of successive beat‐to‐beat interval differences (RMSSD, measured in milliseconds) was calculated as a measure of vmHRV. This time‐domain index reflects the beat‐to‐beat variance in IBI and is less affected by respiratory influences compared to frequency‐domain indexes (Laborde et al., 2017). Then, a recommended coefficient of variation (cv) was applied to obtained “adjusted” cvRMSSD values according to the formulae cvRMSSD = (RMSSD / IBI) × 100 (de Geus et al., 2019).

### 2.2 Statistical analyses

Statistical analyses were conducted using SPSS (version 28, IBM Chicago, IL, USA). The normal distribution of variables was checked by means of the Kolmogorov–Smirnov test. Significantly skewed variables, including RMSSD and cvRMSSD, were log‐transformed (ln) to fit assumptions for linear analyses.

First, participants in both age groups were stratified into two sub‐groups based on their reported sex. ANOVAs were used to determine age‐ and sex‐specific differences in mean IBI and RMSSD values. Zero‐order correlations (Pearson's *r*) were used separately for each sex and age group to assess the relationship between IBI and RMSSD (“adjusted” and not “adjusted”). Correlation coefficients were compared to explored potential age‐ and sex‐specific differences using Fisher's *r*‐to‐z transformation (Steiger, 1980). In order to test if sex moderated the relationship between RMSSD and IBI within each age group, the SPSS macro PROCESS was used (Hayes, 2012). In the program PROCESS, “Model 1” was used to test the interactive effects of RMSSD (“adjusted” and not “adjusted”, independent variable) and sex (moderator; 1 = women 2 = men) on IBI (dependent variable, see Figure 1). We also tested if age moderated the association between RMSSD and IBI within each sex. Specifically, “Model 1” was used to test the interactive effects of RMSSD (“adjusted” and not “adjusted”, independent variable) and age (moderator; 1 = young 2 = old) on IBI (dependent variable, see Figure 1). We also used “Model 2” with both age and sex as moderators in the relationship between RMSSD and IBI in the full sample (Figure 1). Results were similar to those obtained with “Model 1”; thus, only the latter are reported. Johnson‐Neyman region of significance tests were used to determine how men and women differ in IBI at low, mean, and high levels of RMSSD within each age group, and how younger and older individuals differ in IBI at low, mean, and high levels of RMSSD within each sex. High and low values for the predictor variables were derived using ±1SD from the mean. Lastly, we repeated these analyses to examine group differences between older women with and without HRT (Figure 1). All tests controlled for body mass index, and results were identical when considering this covariate. Significance levels were evaluated using an alpha of.0.05.

## 3 RESULTS

### 3.1 Sex and age differences in IBI and RMSSD values

**FIGURE 2:**
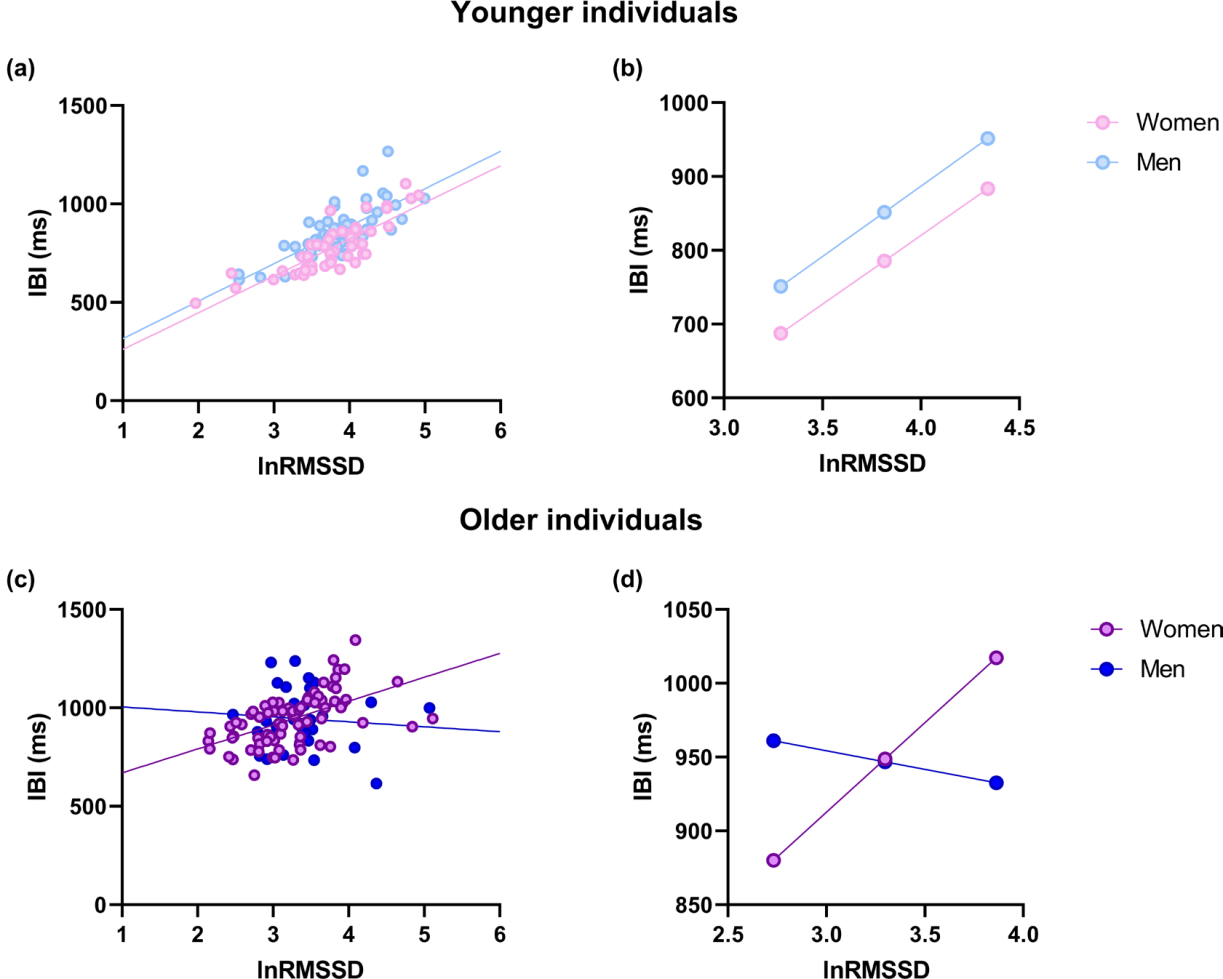
Sex differences in the association between vagally‐mediated heart rate variability and cardiac chronotropy in younger (top panels) and older (bottom panels) individuals. (a) and (c) show scatterplots of lnRMSSD and IBI in men and women of both age groups. (b) and (d) represent the prediction of IBI values in men and women of both age groups at low, mean, and high lnRMSSD values. Higher and lower estimates of lnRMSSD were derived from ±1SD from the mean. IBI, inter‐beat intervals; LnRMSSD, natural log‐transformed root mean square of successive beat‐to‐beat interval differences.

Mean and standard deviations for IBI and RMSSD values are reported in Table 1 and depicted in Figure 2. Younger women showed significantly shorter IBI compared to younger males, with a medium effect size (Cohen's *d* = 0.595). No significant sex differences were found in RMSSD and cvRMSSD values in young individuals. In older individuals, IBI and RMSSD were similar between men and women. However, in both sexes, older individuals showed significantly higher IBI and lower RMSSD values compared to younger individuals. The magnitude of the age‐dependent increase in IBI was large in women (Cohen's *d* = 1.264) and medium in men (Cohen's *d* = 0.671), while the magnitude of the age‐dependent decrease in RMSSD values was overall large in both sexes (Cohen's *d* for RMSSD: women *d* = −0.747 vs. men *d* = −0.680; Cohen's *d* for lnRMSSD: women *d* = −0.887 vs. men *d* = *d* = −0.970; Cohen's *d* for cvRMSSD: women *d* = −1.188 vs. men *d* = −0.810; Cohen's d for ln(cvRMSSD): women *d* = −1.457 vs. men *d* = −1.260).

**TABLE 1:** IBI and RMSSD mean values stratified by age and sex.

	Younger individuals		*p*	Older individuals		*p*	Age comparisons	
	Women	Men		Women	Men		*p*‐women	*p*‐men
IBI (ms)	780.5 ± 130.0	855.7 ± 123.2	**0.003**	945.3 ± 130.6	945.1 ± 149.4	0.995	**<0.001**	**0.002**
RMSSD (ms)	51.1 ± 27.4	51.6 ± 24.8	0.909	31.8 ± 24.8	34.1 ± 27.3	0.675	**<0.001**	**0.002**
lnRMSSD	3.79 ± 0.58	3.84 ± 0.48	0.640	3.27 ± 0.59	3.36 ± 0.52	0.415	**<0.001**	**<0.001**
cvRMSSD	6.25 ± 2.47	5.87 ± 2.29	0.448	3.30 ± 2.49	3.75 ± 3.12	0.401	**<0.001**	**<0.001**
ln(cvRMSSD)	1.75 ± 0.45	1.70 ± 0.38	0.627	1.03 ± 0.52	1.13 ± 0.56	0.334	**<0.001**	**<0.001**

*Note*: Data are reported as means ± standard deviations. Bold values denote statistical significance (*p* < 0.05).

Abbreviations: cv, coefficient of variation; IBI, inter‐beat‐intervals; ln, natural logarithm; RMSSD, root mean square of successive beat‐to‐beat interval differences.

### 3.2 Correlation coefficients between IBI and RMSSD values

Zero‐order correlation coefficients between IBI and RMSSD values are reported in Table 2. In younger individuals, both women and men showed significant positive correlations between IBI and lnRMSSD or ln(cvRMSSD). In older individuals, higher IBI was significantly associated with higher lnRMSSD or ln(cvRMSSD) in women, whereas in men IBI did not significantly correlate with lnRMSSD and was significantly negatively correlated with ln(cvRMSSD). Confirming previous findings (Williams et al., 2022), correlation coefficients between cvRMSSD and IBI were smaller than associations with unadjusted RMSSD values, with the only exception being older men who showed a surprisingly stronger (and negative) correlation between cvRMSSD and IBI compared with unadjusted RMSSD values.

**TABLE 2:** Correlation coefficients stratified by sex and age.

Younger women	1	2	3	Younger men	1	2	3
1. IBI	‐			1. IBI	‐		
2. lnRMSSD	**0.834** **	‐		2. lnRMSSD	**0.740** **	‐	
3. ln(cvRMSSD)	**0.715** **	**0.982** **	‐	3. ln(cvRMSSD)	**0.560** **	**0.971** **	‐

Older women	1	2	3	Older men	1	2	3
1. IBI	‐			1. IBI	‐		
2. lnRMSSD	**0.545** **	‐		2. lnRMSSD	−0.009	‐	
3. ln(cvRMSSD)	**0.346** **	**0.975** **	‐	3. ln(cvRMSSD)	**−0.370** *	**0.975** **	‐

*Note*: Bold *r* values denote statistical significance.

Abbreviations: cv, coefficient of variation; IBI, inter‐beat intervals; ln, natural logarithm; RMSSD, root mean square of successive beat‐to‐beat interval differences.

* *p* < 0.05.

** *p* < 0.01.

### 3.3 Sex differences in the association between RMSSD and IBI in younger and older individuals

Sex differences in the association between RMSSD and IBI in both age groups are depicted in Figure 2.

In young individuals, women showed a stronger correlation between lnRMSSD and IBI than men (Figure 2a). Albeit not significant (*p* = 0.106), the magnitude of such difference (Cohen's *q* = 0.251, small effect) was similar to that found in a previous study in a much larger sample of young individuals (*p* = 0.005 and Cohen's *q* = 0.225 in Williams et al., 2022).

The Johnson‐Neyman technique showed that young women have significantly shorter IBI than men at low, mean, and high lnRMSSD (*p* < 0.05 for all) (Figure 2b). According to the current data, in young individuals with lnRMSSD under approximately 4.36, women showed shorter IBI than men (*p* < 0.05), which is similar to the cut‐off (lnRMSSD = 4.06) previously identified in a larger sample of young individuals (Williams et al., 2022).

In older individuals, sex moderated the association between lnRMSSD and IBI (*R* ^2^_∆_ = 0.068, *B* = 146.5 (49.4), [48.6, 244.4], *p* = 0.004), such that a significant positive association was found in women (*p* < 0.01), but not in men (*p* = 0.559) (Figure 2c). The Johnson‐Neyman technique showed that in older individuals with lnRMSSD under approximately 2.61, women showed shorter IBI than men (*p* < 0.05), while in older individuals with lnRMSSD over approximately 3.86, women showed longer IBI than men (*p* < 0.05) (Figure 2d). Sex also moderated the association between cvRMSSD and IBI (*R* ^2^_∆_ = 0.114, *B* = 185.4 (50.3), [85.7, 285.1], *p* < 0.001).

### 3.4 Age differences in the association between RMSSD and IBI in women and men

**FIGURE 3:**
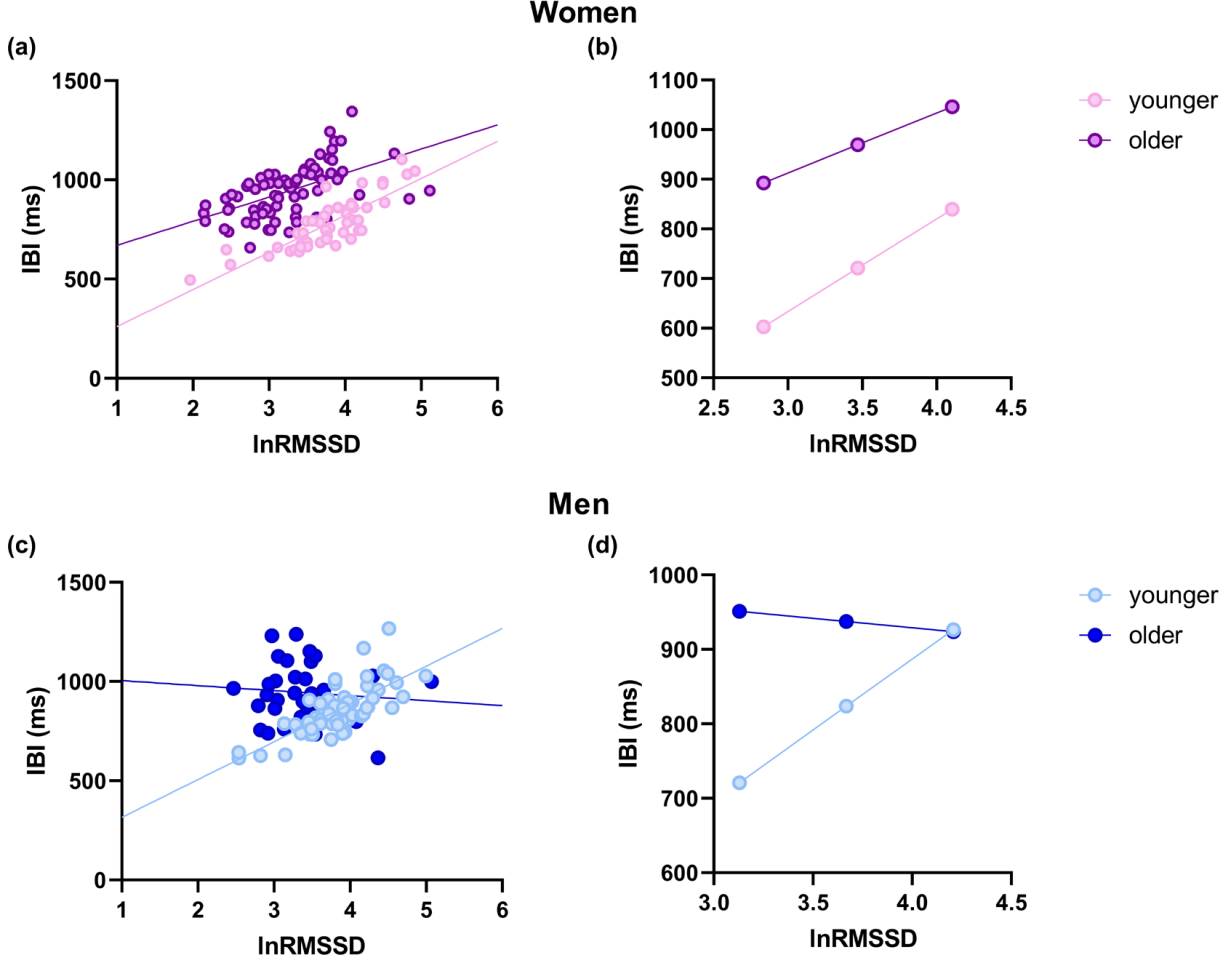
Age differences in the association between vagally‐mediated heart rate variability and cardiac chronotropy in women (top panels) and men (bottom panels) individuals. (a) and (c) show scatterplots of lnRMSSD and IBI in younger and older individuals of both sex groups. (b) and (d) represent the prediction of IBI values in younger and older individuals of both sex groups at low, mean, and high lnRMSSD values. Higher and lower estimates of lnRMSSD were derived from ±1SD from the mean. IBI, inter‐beat intervals; LnRMSSD, natural log‐transformed root mean square of successive beat‐to‐beat interval differences.

Age differences in the association between RMSSD and IBI in both sexes are depicted in Figure 3.

In women, age moderated the association between lnRMSSD and IBI (*R* ^2^_∆_ = 0.015, *B* = −65.3 (31.1), [−127.0, −3.4], *p* = 0.038), such that younger women showed a stronger association than older women (Figure 3a). The Johnson‐Neyman technique showed that younger women have significantly shorter IBI than older women at low, mean, and high lnRMSSD (*p* < 0.05 for all) (Figure 3b). Specifically, in women with lnRMSSD under approximately 4.96, younger individuals showed shorter IBI than older individuals (*p* < 0.05). Likewise, age moderated the association between cvRMSSD and IBI (*R* ^2^_∆_ = 0.033, *B* = −121.6 (44.2), [−209.1, −34.1], *p* = 0.007).

In men, age moderated the association between lnRMSSD and IBI (*R* ^2^_∆_ = 0.137, *B* = −215.6 (49.8), [−314.6, −116.6], *p* < 0.001), such that a significant positive association was found in younger individuals (*p* < 0.001), but not in older individuals (*p* = 0.519) (Figure 3c). The Johnson‐Neyman technique showed that younger men have significantly shorter IBI than older men at low and mean lnRMSSD (*p* < 0.05 for all) (Figure 3d). Specifically, in men with lnRMSSD under approximately 3.84, younger individuals showed shorter IBI than older individuals. Likewise, age moderated the association between cvRMSSD and IBI (*R* ^2^_∆_ = 0.203, *B* = −280.5 (55.8), [−391.5, −169.6], *p* < 0.001).

### 3.5 Differences between older women with or without HRT

**FIGURE 4:**
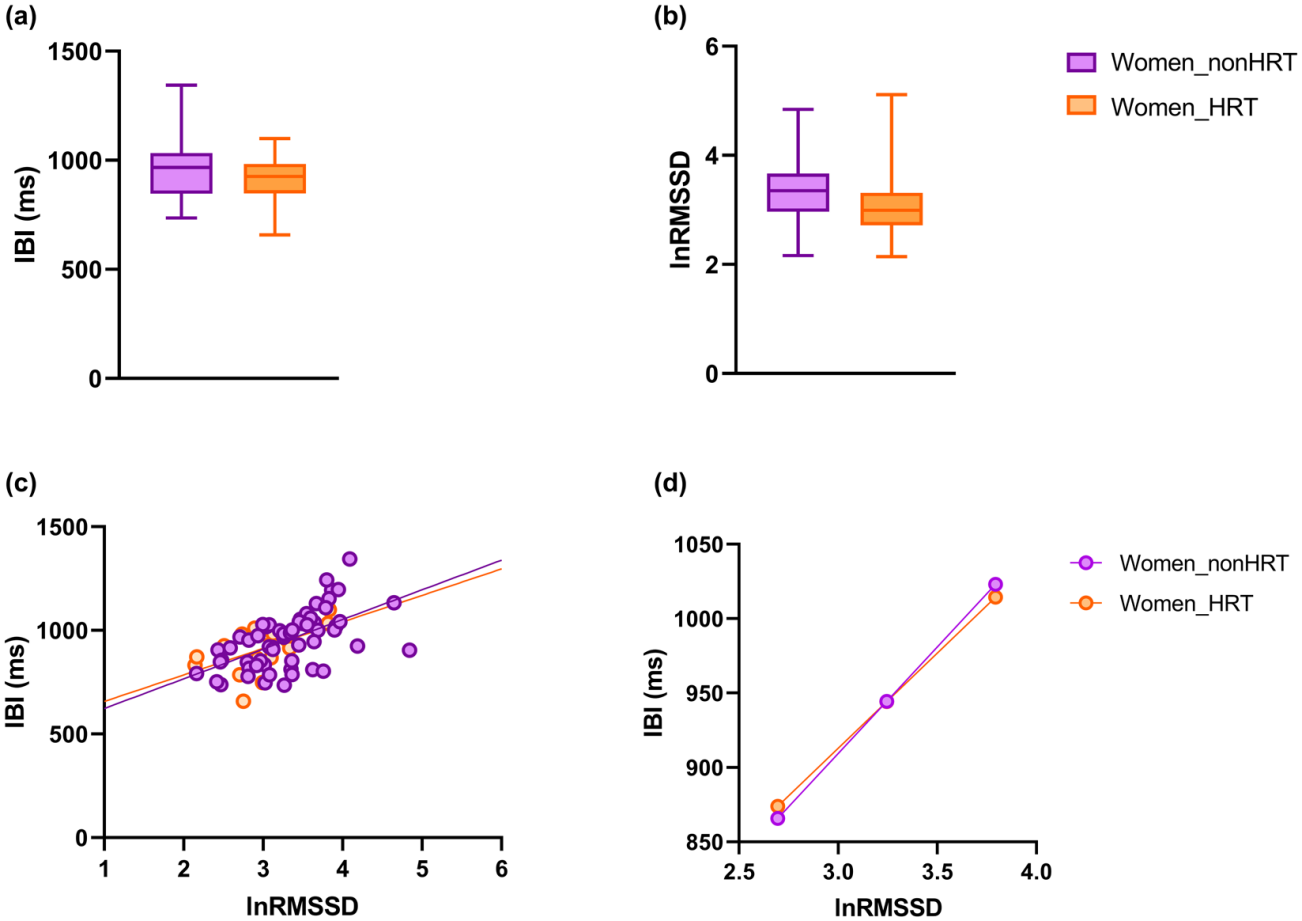
The top panels represent box and whisker plots for IBI (a) and lnRMSSD (b) values in older women with (*n* = 17) or without (*n* = 59) HRT (*n* = 59). The boxes are determined by the 25th and 75th percentiles. The whiskers are determined by 1.5 interquartile range. The bottom panels show a scatterplot of lnRMSSD and IBI values (c) and the prediction of IBI values at low, mean, and high lnRMSSD values (d) in older women with or without HRT. Higher and lower estimates of lnRMSSD were derived from ±1SD from the mean. HRT, hormone replacement therapy; IBI, inter‐beat intervals; lnRMSSD, natural log‐transformed root mean square of successive beat‐to‐beat interval differences.

Differences in mean IBI and RMSSD values and in their association between older women with or without HRT are depicted in Figure 4. There were no differences between the two groups in terms of mean IBI and RMSSD values (Figure 4a,b), as well as in the association between IBI and RMSSD values (Figure 4c,d).

## 4 DISCUSSION

This study replicates and extends previous research on the relationship between measures of vmHRV and cardiac chronotropy by showing (i) stronger associations in young adult and post‐menopausal women compared to age‐matched men, (ii) a weaker or no association in older women and men, respectively, and (iii) no effects of hormone replacement therapy in post‐menopausal women.

A previous study in a large sample (*n* = 663) of young adult individuals (mean age = 19 years) indicated important sex differences in the relationship between indexes of vmHRV (including RMSSD) and cardiac chronotropy (including IBI) (Williams et al., 2022). The current results in another group (*n* = 106) of young adults of the same age range replicate the magnitude of such differences. Specifically, women showed stronger correlations between vmHRV (indexed by RMSSD) and IBI compared to age‐matched men. Of note, correlation coefficients were smaller in both sexes considering “adjusted” RMSSD instead of “unadjusted” values, which is consistent with previous findings (Williams et al., 2022). Also, in this sample of young adults, the average IBI was shorter in women, which is in line with meta‐analytic evidence (Koenig & Thayer, 2016). Notably, Johnson‐Neyman results suggest that at any given level of HRV, young adult women show shorter IBI compared to age‐matched men, supporting the previous findings (Williams et al., 2022). Together, these results are an important confirmation of the differential association between vmHRV and cardiac chronotropy based on sex in young adult individuals.

Both neural and physiological mechanisms have been hypothesized to explain these sex differences. At the neural structural level, the cortical thickness of 14 brain regions has been associated with vmHRV, but not heart rate, in a pooled mega‐analysis which included 1218 individuals (Koenig et al., 2021). Notably, among these brain regions, sex differences emerged in the cortical thickness of the left and right caudal anterior cingulate cortex (thinner in females), left and right insula (thicker in females), left lateral orbitofrontal cortex (thicker in females), and left medial orbitofrontal cortex (thicker in females) (Koenig et al., 2021). Therefore, sex differences in cortical thickness may explain sex differences in cardiac vagal modulation. From a physiological standpoint, stimulation of the vagus nerve elicits the release of ACh, which binds to muscarinic receptors to increase the heart period and HRV. Importantly, converging human and animal evidence suggests that vagal stimulation induces greater pre‐ and post‐synaptic cardiac effects in females compared to males (Du et al., 1994; Huikuri et al., 1996; Pinto et al., 1997; Sarabi et al., 1999; Taddei et al., 1996). These sex differences were not observed in response to methylcoline, a non‐selective muscarinic receptor agonist, suggesting a greater release of ACh by vagal stimulation in female hearts (Du et al., 1994).

Sex differences have also been described within the intrinsic cardiac nervous system, which includes the network of intracardiac ganglia and interconnecting neurons that (i) receive inputs from both local afferent and extrinsic autonomic (vagal and sympathetic) nerves (Achanta et al., 2020), and (ii) perform very complicated integration in regulating cardiac functions and to some extent sinoatrial node activity (Allen et al., 2018; Cardinal et al., 2009; Pauza et al., 2002, 2014). Specifically, female rats were found to have fewer neurons, a lower packing density, and a reduced distribution than males (Leung et al., 2021). Therefore, sex differences in both Ach release and neurons within the intrinsic cardiac nervous system may explain the differential association between vmHRV and cardiac chronotropy between females and males.

This is the first study to investigate the association of vmHRV and cardiac chronotropy as a function of sex and age by extending the analysis to post‐menopausal women vs. age‐matched men (mean age = 72.8 years) with medicated hypertension. Critically, the synthesis and clearance of neurotransmitters, including Ach, in both the heart and vasculature, are influenced by sex hormones such as estrogen (Dart et al., 2002). With advancing age, there is a physiological reduction in sex hormones, with an abrupt and almost complete cessation of both estrogen and progesterone production in post‐menopausal women (Decaroli & Rochira, 2017; Steger & Peluso, 1987). Also, the aging process is characterized by a decline in both cortical thickness (Koenig & Thayer, 2016) and vmHRV (De Meersman & Stein, 2007), which seems to be independent of sex (Koenig & Thayer, 2016).

Importantly, lower levels of vmHRV are associated with increased cardiovascular morbidity and mortality in older individuals (Tsuji et al., 1994). As expected, older individuals were characterized by a reduction in RMSSD values compared to the younger group, independently of sex. Also, in this sample, the IBI was longer in older individuals, without sex differences. Our novel results indicate that in women the association between vmHRV and IBI was weaker in the older group, and Johnson‐Neyman results suggest that at any given level of vmHRV post‐menopausal women show longer IBI compared to young adult women. Remarkably, older men show no association between vmHRV and cardiac chronotropy. This finding is indicative of important sex differences in how changes in vagal modulation of cardiac chronotropy progress over the life course, which may contribute to differences in cardiac outcomes (Merz & Cheng, 2016). For example, most of the available evidence on heart failure, a clinical syndrome that usually develops in older individuals and is characterized by autonomic imbalance, suggests that women compared to men have an overall better long‐term prognosis (Díez‐Villanueva et al., 2021; Huijts et al., 2013; Moser et al., 2011). The current findings indicate that older individuals of both sexes have reduced vmHRV, but only men are characterized by a loss of the association between vmHRV and cardiac chronotropy. This may be indicative of a worse decline of cardiac vagal control in men with advancing age, which may predispose them to enhanced cardiac vulnerability. Of note, correlation coefficients between “adjusted” RMSSD values and IBI were smaller in older women compared to “unadjusted” values and even negative in men. This indicates that adjusting HRV for the heart period is particularly problematic across different age groups, supporting the idea that routine adjustments to measures of HRV must be carefully considered (Williams et al., 2022).

Lastly, the results of our exploratory analysis in a small sample of post‐menopausal women taking HRT reveal no changes in the association between vmHRV and cardiac chronotropy compared with women not taking HRT. Although preliminary, these results might indicate that (i) sex hormone (e.g., estrogen) depletion is not the only mechanism underlying the decline in cardiac vagal modulation in post‐menopausal women, and (ii) exogenous estrogen in older women may not have the same effects on the synthesis of Ach as endogenous estrogen in younger women. However, we do not have information on the nature (e.g., exogenous estrogen and/or progesterone) and timing of HRT in relation to menopause onset, so further studies with larger samples are needed to investigate the effects of HRT on cardiac autonomic function.

The current results must be interpreted with caution, and several limitations must be acknowledged. First, in our sample of older individuals, we could not determine if and to what extent hypertension medications affected cardiac autonomic parameters. Indeed, we do not have details on the exact medication regimen for each participant, and the number of participants not taking any medication was too small (*n* = 41) for conducting a reliable sensitivity analysis on the effect of medication. Second, estrogen levels vary during the menstrual cycle, but we did not have any menstrual cycle information for the younger women of this sample. Third, we adopted only one index of vmHRV (i.e., RMSSD), but previous studies have shown that results are similar when other vmHRV measures are considered (e.g., Williams et al., 2022). Relatedly, we do not have reliable measures of cardiac sympathetic modulation since HRV indices predominantly reflect vagal influences. Previous studies (e.g., Taylor et al., 2001) have shown that sympathetic activity reduces the IBI and opposes vmHRV over high as well as low breathing frequencies. Notably, there is consistent data in healthy young adults to suggest that males have greater sympathetic control of both the heart and vasculature than females (Dart et al., 2002; Fagius & Wallin, 1993). Yet, young adult women show shorter IBI than age‐matched men. Further, pharmacological inhibition of sympathetic activity with beta‐blockers comparably lowers heart rate and blood pressure in hypertensive female versus male individuals (Wilmes et al., 2023), and both sexes experience age‐related increases in sympathetic vascular control to such an extent that sex differences in sympathetic vascular outflow are no longer observed (Keir et al., 2020; Narkiewicz et al., 2005). Thus, it seems unlikely that sex and age differences in IBI are due to differences in cardiac sympathetic control. Finally, the study was underpowered to consider potential ethnic differences, which have been shown to have implications for vmHRV (Hill et al., 2015).

In conclusion, this study provides evidence of sex and age differences in the association between vmHRV and cardiac chronotropy, providing novel insight into vagal mechanisms of cardiac chronotropic control as a function of sex and age. Our results also underscore the importance of considering key demographics such as age and sex assigned at birth in interpretations of HRV findings and potential clinical implications. Further research is needed to elucidate the biological factors that contribute to sex‐ and age‐related differences in the relationship between vmHRV and cardiac chronotropy, which may inform our understanding of individual vulnerability to cardiac and other psychophysiological outcomes.

## FUNDING INFORMATION

This work was supported, in part, by the National Heart, Lung, and Blood Institute (R01HL126056) and the National Institute on Aging (R03AG063328). Darcianne Watanabe is supported by the National Science Foundation Graduate Research Fellowship Program under Grant No. DGE‐1839285.

## CONFLICT OF INTEREST STATEMENT

The authors declare that they have no competing interests.

## Data Availability

The original data are available from the corresponding author upon reasonable request.
